# Response assessment in daily practice: RECIST and its modifications

**DOI:** 10.1186/1470-7330-14-S1-O35

**Published:** 2014-10-09

**Authors:** Aslam Sohaib

**Affiliations:** 1 Dept. of Radiology, Royal Marsden Hospital, London & Sutton, UK

## 

Response Evaluation Criteria for Solid Tumours (RECIST) were introduced in 2000 to provide a standardized method for assessing response to treatments in the clinical trial setting [1]. The RECIST Working Group has updated RECIST to version 1.1 [2]. The revised version has maintained assessment of tumour burden using sum of the diameters and continues to use uni-dimensional measurements. The response categories are still those of complete response, partial response (30% decrease in sum from baseline), stable disease and progressive disease (20% increase in sum from nadir). The revision addressed issues that had arisen related to the use of the criteria in practice.

Though the RECIST criteria are intended for use in the clinical trial setting, oncologists increasingly rely on RECIST based measurements to make clinical management and therapeutic decisions in daily clinical practice. The reasons for this include the clinician’s application of trial data into routine clinical practice but most importantly RECIST offers a simple way of measuring and communicating response assessment. The terms such as measureable disease, tumour burden, target lesions, and response categories are now used and understood universally. RECIST criteria also provide guidance on technique as well as measuring lesions and application to assessing sites of disease, eg lymph node and bones. This allows for a more robust and reproducible way of gauging response. The limitations of RECIST are also well known thereby allowing the clinician to understand the pitfalls in those cases. Therefore the radiologist has to understand and use “RECIST” in their daily practice.
